# Identification of Novel Loci and Candidate Genes for Resistance to Powdery Mildew in a Resequenced Cucumber Germplasm

**DOI:** 10.3390/genes12040584

**Published:** 2021-04-16

**Authors:** Xiaoping Liu, Xingfang Gu, Hongwei Lu, Panna Liu, Han Miao, Yuling Bai, Shengping Zhang

**Affiliations:** 1 Institute of Vegetables and Flowers, Chinese Academy of Agricultural Sciences, Beijing 100081, China; xiaopingliu0@163.com (X.L.); guxingfang@caas.cn (X.G.); lhw516@126.com (H.L.); liupn88@126.com (P.L.); miaohan@caas.cn (H.M.); 2 Plant Breeding, Wageningen University & Research, Droevendaalsesteeg 1, 6708 PB Wageningen, The Netherlands

**Keywords:** cucumber, GWAS, polygenic, powdery mildew, recessive

## Abstract

Powdery mildew (PM) is one of the most serious diseases in cucumber and causes huge yield loss. Multiple quantitative trait loci (QTLs) for PM resistance have been reported in previous studies using a limited number of cucumber accessions. In this study, a cucumber core germplasm (CG) consisting of 94 resequenced lines was evaluated for PM resistance in four trials across three years (2013, 2014, and 2016). These trials were performed on adult plants in the field with natural infection. Using genome-wide association study (GWAS), 13 loci (*pmG1.1*, *pmG1.2*, *pmG2.1*, *pmG2.2*, *pmG3.1*, *pmG4.1*, *pmG4.2*, *pmG5.1*, *pmG5.2*, *pmG5.3*, *pmG5.4*, *pmG6.1*, and *pmG6.2*) associated with PM resistance were detected on all chromosomes except for Chr.7. Among these loci, ten were mapped to chromosomal intervals where QTLs had been reported in previous studies, while, three (*pmG2.1*, *pmG3.1*, and *pmG4.1*) were novel. The loci of *pmG2.1*, *pmG5.2*, *pmG5.3* showed stronger signal in four trials. Based on the annotation of homologous genes in Arabidopsis and pairwise LD correlation analysis, candidate genes located in the QTL intervals were predicted. SNPs in these candidate genes were analyzed between haplotypes of highly resistant (HR) and susceptible (HS) CG lines, which were defined based on combing disease index data of all trials. Furthermore, candidate genes (*Csa5G622830* and *CsGy5G015660*) reported in previous studies for PM resistance and cucumber orthologues of several PM susceptibility (S) genes (*PMR5*, *PMR-6*, and *MLO*) that are colocalized with certain QTLs, were analyzed for their potential contribution to the QTL effect on both PM and DM in the CG population. This study shows that the CG germplasm is a very valuable resource carrying known and novel QTLs for both PM and DM resistance, which can be exploited in cucumber breeding.

## 1. Introduction

Cucumber (*Cucumis sativus* L.) is an important vegetable crop with a long history of cultivation. Powdery mildew (PM), caused mainly by the fungus *Podosphaera fusca* is one of the most important diseases in cucumber and occurs extensively in various countries all over the world [1,2,3,4]. PM is a foliar disease and generally occurs in the middle and late growth period of cucumber production although it can occur during the whole growth season. At the early stage of the infection, white, small round powdery fungal colonies appear on the top of the leaves, which gradually expand, leading to the joining of individual colonies. At the later stage, leaf areas underneath powdery colonies turn chlorotic and the infected leaves gradually shrink. Finally, the infected leaves lose photosynthetic function, leading to prematurely aged and dead. Due to the decrease on foliage and photosynthetic efficiency, yield from PM infected plants are reduced.

Development of resistant cultivars is the most economic and effective method to control PM in cucumber. In the 1940s and 1950s, PM resistance was identified in Asian germplasms. For example, the PR37 line derived from Chinese germplasm; Yomaki (PI 288,238) and cv. Natsufushinari from Japan, as well as PI 197,087, cv. Bangalore and Burma lines (PI 200,815 and PI 200,818) from India (Table 1). Resistance in these accessions or old cultivars were shown to be incomplete and inherited as a polygenic and/or recessive trait [1,2,3,4]. These resistant cultivars/accessions and their derived lines were the starting points for cucumber PM resistance breeding. For example, the NPI line that was generated from crosses between Natsufushinari and Burma lines was frequently used in the Dutch breeding program [3]. Later in 1992 and 2005, two screenings for PM resistance were carried out in 177 and 977 cucumber accessions, respectively [5,6]. Most of these accessions were from Asian germplasm, except a few from Europe that were breeding lines with PM resistance introgressed likely from Asian lines such as the NPI line. Incomplete PM resistance was identified in a limited number of accessions and inheritance of the resistance was described again as recessive and polygenic based on the segregation ratio in F1s and F2s [7].

With the advent of molecular markers in the 1980s, it became feasible to map polygenic traits to chromosomal regions, spanning an interval defined by two or more molecular makers that are significantly correlated with the evaluated trait such as disease resistance [8]. In 2006, the first quantitative trait locus (QTL) mapping was carried out to study the PM resistance in accession PI 197,088 (from India and highly resistant) and cv. Santou (from Japan, partial resistant) by using a set of recombinant inbred lines (RILs) [9,10]. This was followed by many other studies resulting in many detected QTLs for PM resistance that are scattered over all the seven cucumber chromosomes [3,11,12,13,14,15] (Table 1). For some chromosomes, more than one QTL derived from one or different resistance donors are clustered. For example, three QTLs (*pm5.1*, *pm5.2*, *pm5.3*) are located on chromosome 5 that are reported in different studies [11,16,17,18,19,20,21,22]. Further fine-mapping is needed to narrow down the QTL interval in order to pinpoint the causal genes for each QTL.

**Table 1:** Overview of mapped and cloned genes/quantitative trait loci (QTLs) for powdery mildew resistance in cucumber.

Resistance Donor	Mapping Population	Gene/QTL	Chromosome	Causal Gene	Reference
Puerto Rico 37	-	-	-	-	[14]
Indian accession PI197087	-	-	-	-	[15]
Yomaki	-	-	-	-	[13]
Natsufushinari	-	-	-	-	[3]
Indian accession PI 197088-1	RILs of PI 197088-1 × Santou	-	1, 5, 6, 7		[12]
S94	RILs (F_6:7_ family) of Inbred line S94 (R) × S06(S)	*pm1.1*, *pm2.1*, *pm4.1*, *pm6.1*	1, 2, 4, 6		[9]
WIS2757	F_2_ family of WIS2757 × 19032	-	5		[20]
K8	F_2_ and F_2:3_ families of K8 (R) × K18 (S)	*pm5.1*, *pm5.2*, *pm5.3*, *pm6.1*	5, 6	-	[11]
WI 2757	F_2:3_ families of WI2757 (R) × True lemon (S)	*pm1.1*, *pm1.2*, *pm3.1*, *pm4.1*, *pm 5.1*, *pm5.2*	1, 3, 4, 5	-	[17]
CS-PMR1	RILs of CS-PMR1 (derived from PI 197088, R) × Santou (intermediate S)	9 QTLs	All chromosomes except 7	-	[22]
S1003	BC3F1 and BC2F2 families of S1003(R) × S1001(S)	*pm5.1*	5	*CsaMLO8* (*CsMLO1*)	[18,23]
Jin5-508	F_2_ and CSILs families of Jin5-508(R) × D8(S)	*pm1.1*	1		[24]
NCG122	F_2_ families of NCG122 (R) × NCG121(S)	*pm-s*	5		[10]
PI 197088	RIL families of PI 19,788 (R) × Coolgreen (S)	*pm1.1*, *pm2.1*, *pm5.1*, *pm 6.1*	1, 2, 5, 6		[21]
IL52	RIL families of IL52 (R) × changchunmici (S)	*pm*	5	*Csa5M622830*	[25]
PM-R	F_2_ families of PM-R × PM-S	*pm5.2*, *pm6.1*	5, 6	*CsGy5G015660*	[16]

Till now, a few candidate genes have been predicated to be the candidate genes underlying PM QTLs (Table 1). In the study of Xu et al. [24], fine-mapping a QTL on chromosome 1 led to discovery of two potential candidate genes, *Csa1M064780* and *Csa1M064790*, both encoding receptor-like kinases with cysteine-rich extracellular domains. This QTL is derived from a northern China genotype Jin5-508. In the study of Zhang et al. [25], the gene *Csa5M622830* was reported to be likely the candidate gene for the completely PM resistance introgressed from *Cucumis hystrix* [25], which encodes a GATA transcriptional factor. For the QTL in one Korean breeding line [16], the *CsGy5G015660* gene was suggested to be the causal gene, which encodes a putative leucine-rich repeat receptor-like serine/threonine-protein kinase (RPK2).

Most of the QTLs showed recessive inheritance, indicating that the resistance is governed by loss-of-function mutant alleles of certain susceptible genes [26,27]. Indeed, a mutant allele of the cucumber susceptibility gene *CsaMLO8* [28] (named as *CsMLO1* in [23]) explains the contribution to the hypocotyl resistance to PM by the QTL *pm5.1* derived from the accession PI 197,088 [28]. In additional to hypocotyl resistance, this QTL was suggested to be necessary for complete or leaf resistance [3,11,12,13,14,15].

Susceptibility genes (S-genes) is an alternative strategy for achieving durable resistance [26]. In van Schie and Takken’s review, more than 180 S-genes were identified in various plant species [29]. Among these S-genes, several have been proved to be functional S-genes for PMs, such as *MLO* (*Mildew Locus O*)-like genes and *PMR* (*powdery mildew resistant*) genes for which cucumber orthologs were identified and mapped to chromosomes [27]. Some of the S-genes colocalize with QTLs for PM resistance, which may indicate that mutations in certain S-genes explain the QTL effect. This was proven to be true for the loss-of-function mutation in *CsaMLO8* that contributes to one QTL for hypocotyl resistance [28].

In this study, 94 core germplasm (CG) lines [30] were used for a GWAS on PM resistance. In total, 13 QTLs were detected, of which three are novel and ten were reported in previous studies. Further, potential candidate genes for certain QTLs were studied by evaluating their expression in contrast lines with high resistance or susceptibility. Allelic variations were analyzed for cucumber orthologs of certain S-genes that are located next to or in QTL intervals as well as the candidate genes identified in previous studies [13,18,19,20,21,22,24,25].

## 2. Materials and Methods

### 2.1. Plant Materials

A cucumber CG population consisting of 94 lines (Supplementary File S1) was provided by the cucumber research group in the Institute of Vegetables and Flowers, Chinese Academy of Agricultural Sciences, China. This CG population was selected from more than 3000 germplasms worldwide and resequenced [30]. Sequencing data can be found on the NCBI Short Read Archive (SRA) under accession SRA056480 [30].

### 2.2. Disease Tests and Disease Index Calculation

The phenotyping of PM resistance was carried out in the greenhouse of Nankou farm (40°13′ N, 116°09′ E) and Shunyi farm (40°15′ N, 116°83′ E) in Beijing from 2013 to 2016. Disease tests with PM were performed four times, in Spring 2013 (pm_2013S), Spring 2014 (pm_2014S), Autumn 2014 (pm_2014A), and Spring 2016 (pm_2016S). In each test, randomized block design was applied with three blocks and six plants per line were used for each block.

Naturally PM infection occurred at the adult plant stage. PM appeared about four weeks after sowing. From five weeks after sowing, PM symptoms was scored once a week for three weeks by ranking the disease of each plant with 0, 1, 3, 5, 7, and 9, where 0: no symptom; 1: ≤1/3th of all leaves with PM spots; 3: 1/3–2/3 of all leaves with PM spots; 5: ≥2/3 of all leaves with PM spots; 7: PM spots covers the whole leaf; 9: ≥2/3 of the browning leaves [13]. For each line, a disease index (DI) was calculated for each line per experiment. DI = 100 × ∑(Number of plants with disease rating × Disease rating)/(Total number of plants × Highest disease rating) [31].

### 2.3. Genetic Diversity of PM Resistance in the CG Population

To cluster the CG lines, a phylogenetic tree was constructed based on their responses to PM infection using SAS 9.0 based on the average DI of each CG line in four experiments [32].

### 2.4. Genome-Wide Association Analyses of PM Resistance

FastLMM was used for association tests of resistance with an estimated relatedness matrix as covariate. GWAS was conducted and the genome-wide lowest *p* value was recorded. The 5% lowest tail was taken from the 200 recorded minimal *p* values as the threshold for genome-wide significance. The Manhattan map for GWAS was generated using the R package CMplot (https://github.com/YinLiLin/R-CMplot, accessed on 14 April 2021). The SNP data used for the association analysis was downloaded from the cucumber genome website: http://www.icugi.org/cgi-bin/ICuGI/index.cgi, accessed on 14 April 2021 [30].

### 2.5. Linkage-Disequilibrium (LD) Analysis

The software Plink [33] was used to calculate the LD coefficient (R^2^) between pairwise high-quality SNPs; the parameters were set as: ‘--r2 --ld-window 999,999 --ld-window-kb 1000 --ld-window-r2 0’, and the results were used to estimate LD decay.

### 2.6. Candidate Gene Analysis

The mapped QTLs of this study were compared with published studies to identify the novel ones, for which candidate region was analyzed within 50 kb around peak SNPs. The three strongest candidate regions were analyzed by the position (−log10 (*p*-value) = 5 (−log10 (*p*-value) = 8 in spring 2016) as the critical value). The candidate gene was annotated. The physical distance was based on the cucurbit genomics (Chinese Long genome v2) (http://cucurbitgenomics.org/, accessed on 14 April 2021). The choice of the 50-kb region was based on the fact that LD decay was less than 50 kb in most cucumber materials [30]. SNPs in the CDS region were used to analyze the candidate genes.

## 3. Results

### 3.1. Genetic Diversity of PM Resistance in the CG Population

**Figure 1:**
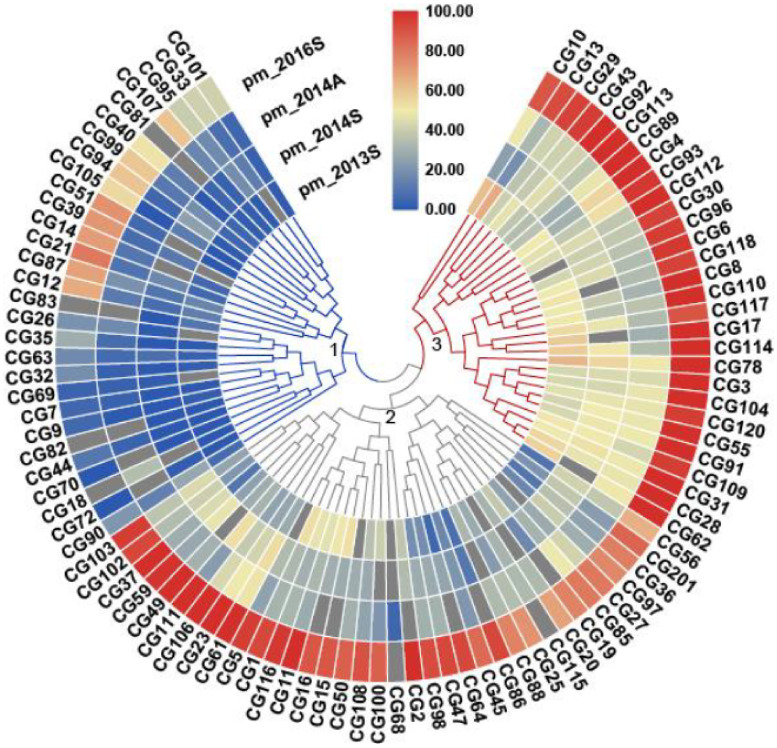
Heatmap depicting the phenotypic distribution of disease index (DI) of powdery mildew (PM) resistance in four environments. The three clusters of the core germplasm (CG) lines are numbered with 1 to 3, and data of mean DI per CG line in the four experiments were used for this clustering analysis. The numbers next to the color key represent DI values. Blue means resistant and red susceptible. The intensity of the color indicates the level of resistance/susceptibility.

**Figure 2:**
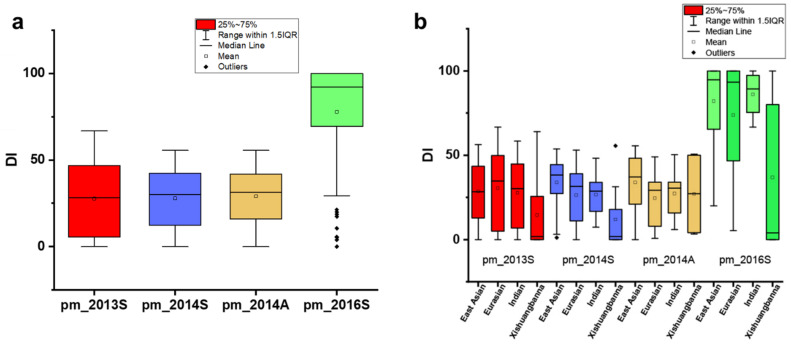
Box plots depicting the phenotypic distribution of disease index (DI) of powdery mildew (PM) resistance in four experiments and among different ecotypes. (**a**) Phenotypic distribution in four experiments; (**b**) DI distribution in four ecotypes.

The 94 CG lines were evaluated for PM resistance four times under natural infection, in spring 2013 (pm_2013S), spring 2014 (pm_2014S), autumn 2014 (pm_2014A) and spring 2016 (PM_2016S). A mean DI was calculated for each line per experiment (Figure 1, Supplementary File S1). Overall, a significant DI correlation (R^2^ = 0.69~0.82, Supplementary Figure S1) was found among the four experiments. The mean DI of pm_2016S was higher than the other three experiments (Figure 2a), indicating that PM was more severe in the spring of 2016. In this CG population, there are four market types of cucumber, including the East Asian type (*n* = 35 CG lines), the Eurasian type (*n* = 29), the Indian type (*n* = 21), and the Xishuangbanna type (*n* = 9) (Figure 2b) (Supplementary File S2). A similar mean DI was found for all market types except for the Xishuangbanna type that had a lowest mean DI in three of four experiments (except for 2014A) (Figure 2b). However, it should be noted that there are a few lines of Xishuangbanna type included in the study.

Based on the average DI of the four experiments, these CG lines could be grouped into three clusters; 1: Resistant; 2: Intermediate resistant, and 3: Susceptible (Figure 1). The disease scores of some lines differed among experiments, which could be due to the fact that the naturally occurring PM infection was possibly not evenly distributed. Thus, for further analysis, all phenotypic data of the four experiments were used to define highly resistant and susceptible lines. A line was considered highly susceptible (HS) if it had a DI above 50 in at least two of the four experiments and highly resistant (HR) line with a DI smaller than 40 in all the experiments (Figure 1, Supplementary File S1). Intermediate resistant (IR) lines were with a DI above 40 in at least one of the four experiments and smaller than 80 in all the experiments. From the tested 94 CG lines, we could define 12 HR and 21 HS lines, in which different ecotypes are presented (Supplementary File S3).

### 3.2. Genetic Loci Associated with PM Resistance in the CG Population

**Figure 3:**
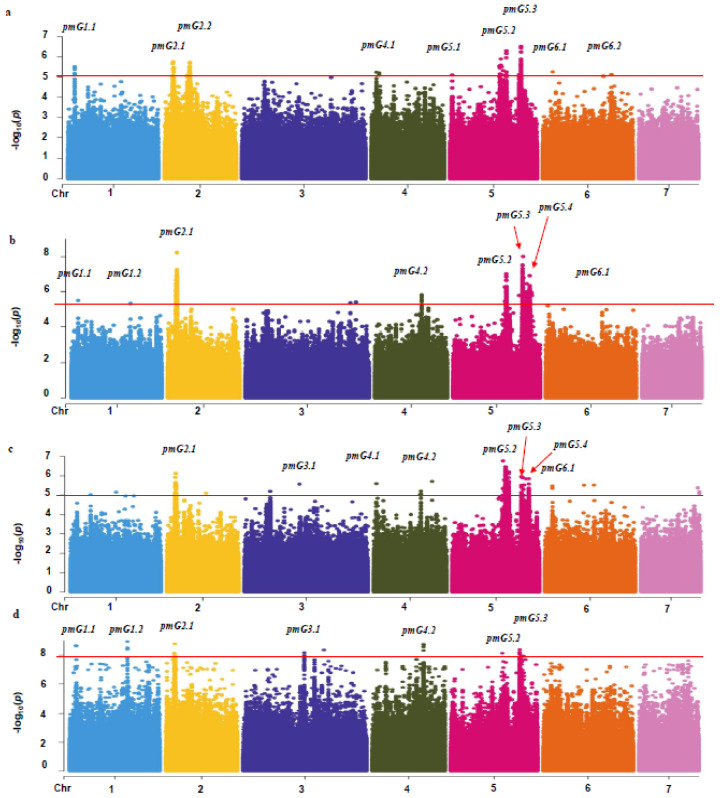
GWAS Mahattan plots of PM resistance in four seasons. (**a**) pm_2013S; (**b**) pm_2014S; (**c**) pm_2014A; (**d**) pm_2016S. The threshold line in 2013 and 2014 is −log_10_(*p*) = 5 and −log_10_(*p*) = 8 in spring 2016.

In order to identify genetic loci that are associated with PM resistance in the CG population, the DI of each experiment was used for GWAS. Thirteen loci were detected, which are scattered on six chromosomes (1 to 6, Figure 3).

**Figure 4:**
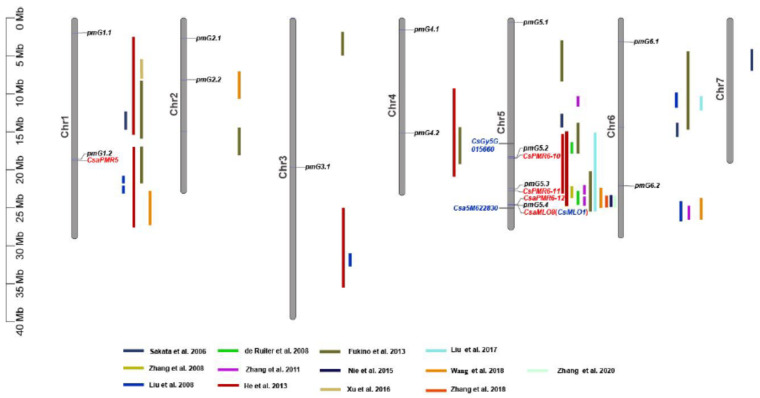
Locations of QTLs for powdery mildew resistance in cucumber reported in this study and previous studies (2006–2020). The black font indicates the loci of this study and their exact SNP locations can be found in (Supplementary File S4), red font indicates the S-genes, blue font indicates candidate gene identified in previous studies.

Three loci, *pmG2.1*, *pmG5.2*, and *pmG5.3*, were detected in all the four experiments, indicating that these three loci were stable related to PM resistance. For the rest, two loci (*pmG1.1* and *pmG4.2*) were detected in three experiments, five loci (*pmG1.2*, *pmG3.1*, *pmG4.1*, *pmG5.4*, and *pmG6.1*) in two experiments and one locus *pmG2.2* in only one experiment. Among the 13 loci, 10 loci were located in chromosomal regions where QTLs for PM resistance were reported in previous studies and three loci (*pmG2.1*, *pmG3.1*, and *4.1*) are novel (Figure 4).

### 3.3. Candidate Genes for PM Resistance

Aiming at identifying potential candidate genes of the novel (*pmG2.1*, *pmG3.1*, *pmG4.1*) and stable loci detected in four experiments (*pmG2.1*, *pmG5.2*, and *pmG5.3*), chromosomal region of 50 kb around the peak SNPs were further analyzed.

**Figure 5:**
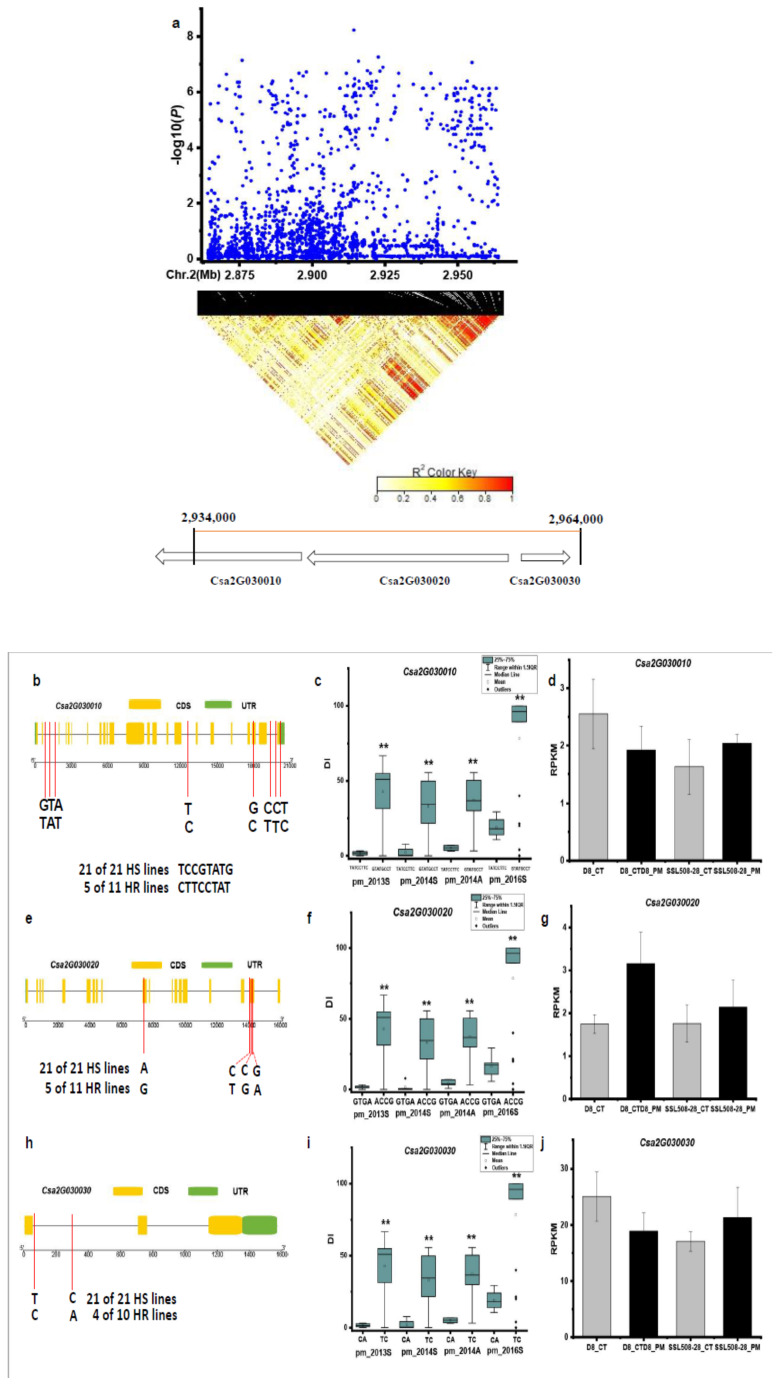
Identification of the causal gene for the locus *pmG2.1*. (**a**) Local Manhattan plot [9] and LD heatmap (bottom) surrounding the peak of *pmG2.1*. (**b**,**e**,**h**) SNP variation of the candidate gene *Csa2G030010*, *Csa2G030020*, and *Csa2G030030* between the highly susceptible (HS) and highly resistant (HR) lines (see Supplementary File S5). (**c**,**f**,**i**) Disease index (DI) comparison between haplotypes of the HS and HR lines for the candidate gene *Csa2G030010*, *Csa2G030020*, and *Csa2G030030*, respectively. (**d**,**g**,**j**) RPKM (Reads Per Kilobase Million) of candidate gene *Csa2G030010*, *Csa2G030020* and *Csa2G030030*, respectively, based on the transcriptome of PRJNA321023 (data obtained from http://cucurbitgenomics.org/rnaseq/home, accessed on 14 April 2021). ** indicates significance at *p* < 0.01.

For the *pmG2.1* locus, SNPs of the chromosome region Chr. 2: 2934–2964 kb were analyzed by pairwise LD correlations with a focus on candidate genes in the interval from 2864 to 2964 kb (Figure 5a). Based on the Cucumber Genome Browser (http://cucurbitgenomics.org, accessed on 14 April 2021), three candidate genes (*Csa2G030010*, *Csa2G030020*, and *Csa2G030030*) are located in this interval. All SNPs in these three genes showed a significant DI difference between haplotypes of the defined HS and HR lines in the four experiments (Figure 5c,f,i). In the *Csa2G030010*, encoding DNA polymerase, five of the eleven HR lines had the CTTCCTAT haplotype, all the 21 HS lines carried the TCCGTATG haplotype (Figure 5b; Supplementary File S5). The expression level of this gene was down-regulated in the susceptible material D8 and up-regulated in the resistant segment substitution line SSL508-28 after 48 h inoculation with PM (Figure 5d). The *Csa2G030020* gene encodes a Lin-9-like protein. With the SNPs identified, five of the eleven HR lines had the GTGA haplotype, all the 21 HS lines carried the ACCG haplotype (Figure 5e; Supplementary File S5). The expression level of this gene was up-regulated in both SSL508-28 and D8 after 48 h inoculation with PM (Figure 5g). Two SNPS were found in the *Csa2G030030* gene encoding an unknown protein. Four of the ten HR lines had the CA haplotype, all the 21 HS lines carried the TC haplotype (Figure 5h; Supplementary File S5). The expression level of this gene was down-regulated in the susceptible material D8 and up-regulated in the resistant segment substitution line SSL508-28 after 48 h inoculation with PM (Figure 5j).

**Figure 6:**
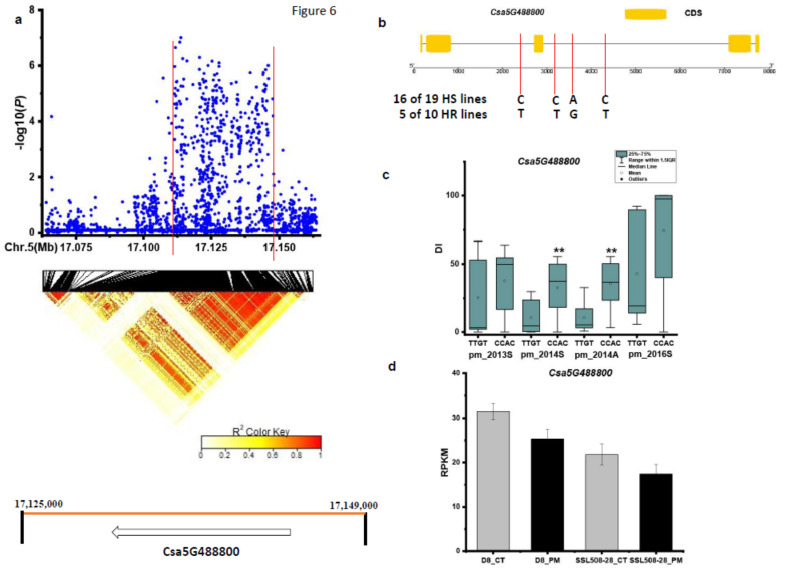
Identification of the causal gene for the locus *pmG5.2*. (**a**) Local Manhattan plot [9] and LD heatmap (bottom) surrounding the peak *pmG5.2*. (**b**) SNP variation of the candidate gene *Csa5G488800* between the highly susceptible (HS) and highly resistant (HR) lines (see Supplementary File S5). (**c**) Disease index (DI) comparison between haplotypes of the HS and HR lines. (**d**) RPKM (reads per kilobase million) of candidate gene *Csa5G488800* based on the transcriptome of PRJNA321023 (data obtained from http://cucurbitgenomics.org/rnaseq/home, accessed on 14 April 2021). ** indicates significance at *p* < 0.01.

For the *pmG5.2* locus, SNPs in the chromosomal region of Chr.5: 17,063–17,163 kb were analyzed (Figure 6a). We focused on the interval from 17,125 to 17,149 kb based on pairwise LD correlations (r^2^ ≥ 0.6) and identified one candidate gene, *Csa5G488800*. SNPs in *Csa5G488800* (RNA recognition motif) showed a significant DI difference between haplotypes of the defined HS and HR in Spring 2014 and Autumn 2014 (Figure 6b,c). In the HS lines, 16 out of the 19 HS lines carried the CCAC haplotype, while 5 out of the 10 HR lines had the TTGT haplotype (Figure 6b). The expression level of this gene was down-regulated in both SSL508-28 and D8 after 48 h inoculation with PM (Figure 6d).

**Figure 7:**
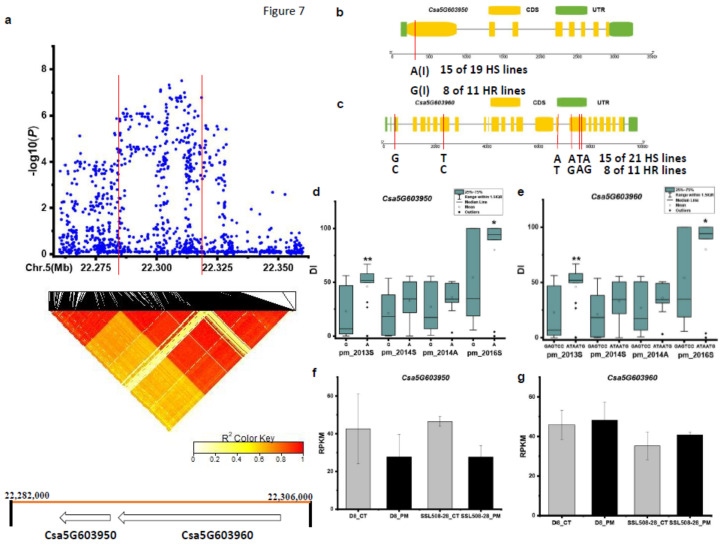
Identification of the causal gene for the locus *pmG5.3*. (**a**) Local Manhattan plot [9] and LD heatmap (bottom) surrounding the peak of *pmG5.3.* (**b**,**c**) SNP variation of the candidate gene *Csa5G603950* and *Csa5G603960* between the highly susceptible (HS) and highly resistant (HR) lines (see Supplementary File S5). (**d**,**e**) Disease index (DI) comparison between haplotypes of the HS and HR lines of *Csa5G603950* and *Csa5G603960*; (**f**,**g**) RPKM (reads per kilobase million) of candidate gene *Csa5G603950* and *Csa5G603960*, respectively based on the transcriptome of PRJNA321023 (data obtained from http://cucurbitgenomics.org/rnaseq/home, accessed on 14 April 2021). (**g**) RPKM (reads per kilobase million) of candidate gene *Csa5G603960* based on the transcriptome of PRJNA321023 (data obtained from http://cucurbitgenomics.org/rnaseq/home, accessed on 14 April 2021); * and ** indicate significance at *p* < 0.05 and *p* < 0.01, respectively.

For the *pmG5.3* locus, SNPs in the chromosomal region of Chr.5: 22,260–22,360 kb was analyzed with the focus on the interval from 22,282 to 22,306 kb using pairwise LD correlations (r^2^ ≥ 0.6) (Figure 7a). Two candidate genes (*Csa5G603950* and *Csa5G603960*) were identified in this interval. SNPs in *Csa5G603950* (Pyruvate dehydrogenase E1 component α subunit) showed a significant DI difference between haplotypes of the defined HS and HR in spring 2013 and spring 2016 (Figure 7d). In the HS lines, 15 out of the 19 HS lines carried the A haplotype, while 8 out of the 11 HR lines had the G haplotype (Figure 7b). SNPs in *Csa5G603960* (paired amphipathic helix protein Sin3) showed a significant DI difference between haplotypes of the defined HS and HR in spring 2013 and autumn of 2016 (Figure 7b). In the HS lines, 15 out of the 21 HS lines carried the GTAATA haplotype, while 8 out of the 11 HR lines had the CCTGAG haplotype (Figure 7b). The expression level of *Csa5G603950* was down-regulated in SSL508-28 and D8 after 48 h inoculation with PM (Figure 7f). While for the *Csa5G603950* gene, a similar expression level before and post inoculation was observed (Figure 7g).

**Figure 8:**
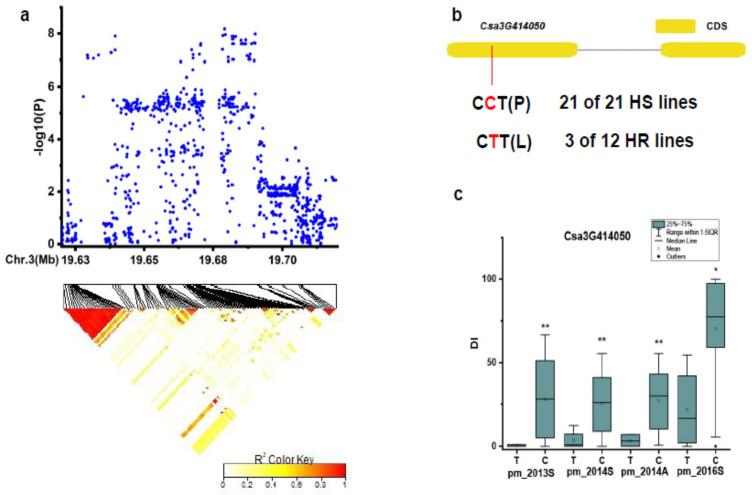
Identification of the causal gene for the locus *pmG3.1*. (**a**) Local Manhattan plot [9] and LD heatmap (bottom) surrounding the peak of *pmG3.1.* (**b**) SNP variation of the candidate gene *Csa3G414050* between the highly susceptible (HS) and highly resistant (HR) lines (see Supplementary File S5). (**c**) Disease index (DI) comparison between haplotypes of the HS and HR lines. * and ** indicate significance at *p* < 0.05 and *p* < 0.01, respectively.

For the novel locus *pmG3.1*, SNPs in the chromosomal region of Chr.3: 19,629–19,729 kb was analyzed (Figure 8a). Only one gene *Csa3G414050* (unknown protein) was identified with one SNP variation in the CDS region (Figure 8b). This SNP showed significance on DIs between haplotypes of the defined HR and HS lines in the four experiments (Figure 8c). In the HR and HS lines, 21 out of the 21 HS lines carried the C(P) haplotype, while 3 out of the 12 HR lines had the T(L) haplotype (Supplementary File S5) (Figure 8b). No expression of *Csa3G414050* was detected in the control and after 48 h inoculation with PM in SSL508-28 and D8.

**Figure 9:**
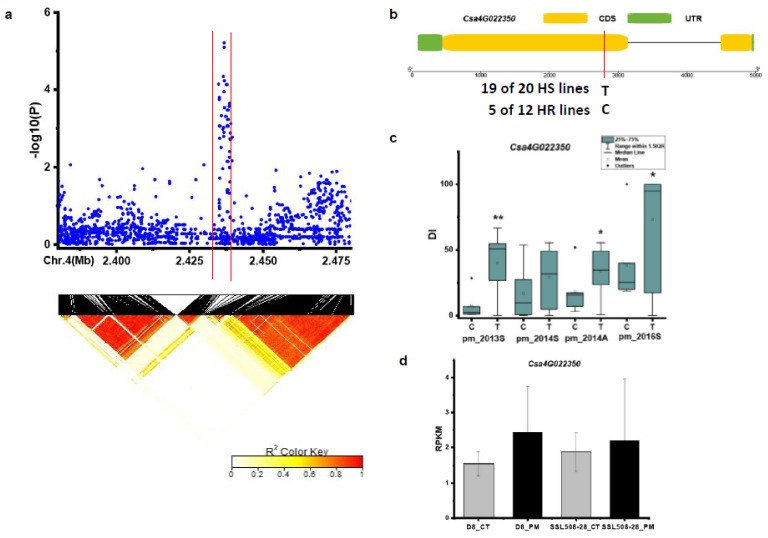
Identification of the causal gene for the locus *pmG4.1*. (**a**) Local Manhattan plot and LD heatmap (bottom) surrounding the peak *pmG4.1*. (**b**) SNP variation of the candidate gene *Csa4G022350* between the highly susceptible (HS) and highly resistant (HR) lines (see Supplementary File S5); (**c**) Disease index (DI) comparison of candidate gene *Csa4G022350* between haplotypes of the HS and HR lines. (**d**) RPKM (reads per kilobase million) of candidate gene *Csa5G603960* based on the transcriptome of PRJNA321023 (data obtained from http://cucurbitgenomics.org/rnaseq/home, accessed on 14 April 2021). * and ** indicate significance at *p* < 0.05 and *p* < 0.01, respectively.

For the novel locus *pmG4.1*, 22 kb region (Chr.4:2458–2480 kb) was obtained from Chr.4: 2386–2486 kb using pairwise LD correlations (r^2^ ≥ 0.6) (Figure 9a). Only on gene *Csa4G022350* (encoding receptor-like protein kinase) was present in this 22 kb region. Based on SNPs in the CDS region of *Csa4G022350* (Figure 9b), one SNP showed significance on DIs between haplotypes of the defined HR and HS lines in the spring 2013, spring 2014, and spring 2016 (Figure 9c). In the HR and HS lines, 19 out of the 20 HS lines carried the T haplotype, while 5 out of the 12 HR lines had the C haplotype (Figure 9b) (Supplementary File S5). The expression level of *Csa4G022350* was up-regulated in SSL508-28 and D8 after 48 h inoculation with PM (Figure 9d).

### 3.4. Haplotype Analysis of Candidate Genes Reported in Literature for PM Resistance/Susceptibility

**Figure 10:**
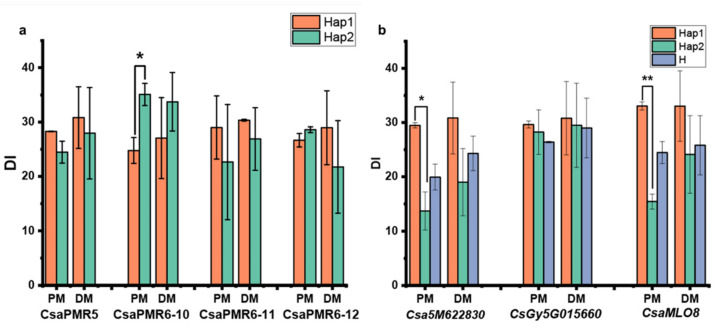
Disease index (DI) of PM and DM in the haplotype of cucumber orthologues of PMR genes and three PM resistance genes identified in previous studies (see haplotypes of each gene in the Supplementary Files S6 and S7). (**a**,**b**) Comparison of DI between haplotypes of the selected candidate genes for PM (average DI of in pm_2013S, pm_2014S, and pm_2014A and for DM (average DI of dm_2014S; dm_2014A; and dm_2015A, the DM data was taken from our previous publication [35]). * and ** indicate significance at *p* < 0.05 and *p* < 0.01, respectively.

In previous studies, a number of genes have been reported that function as susceptibility (S) genes for PM, such as the Clade V *MLO* genes and *PMR* genes, and their cucumber homologues have been identified [27]. Some of these cucumber homologues co-localize with certain QTLs mapped in this and previous studies, including *CasPMR5* with *pmG1.2*, *CsaPMR6-10* with *pmG5.2*, *CsaPMR6-11* with *pmG5.3*, *CsaMLO8* and *CsaPMR6-12* with *pmG5.4* (Figure 4). In order to verify the potential link between these S genes and the QTLs, we analyzed the effect of SNPs in these S genes on PM DI of the CG lines. We also included the DM data of this CG population in our previous study [34] since it has been often reported that resistance to PM and DM is linked in cucumber [21], which is supported by the significant correlation between the DIs of PM and DM in this CG population (Supplementary Figure S2). Based on the SNPs variation in the *PMR* genes (Supplementary File S6), only SNPs in *CsaPMR6-10* showed a significant difference on DI of PM (the average of spring 2013, spring 2014, and autumn 2014), but not on DI of DM (the average of spring 2014, autumn 2014, and autumn 2015) (Figure 10a).

It has been shown that loss-of-function of the cucumber *CsaMLO8* gene leads to the hypocotyl PM resistance in cucumber [23,28]. In the study of Berg et al. [28], a transposable element (TE) insertion was found in *CsaMLO8*, resulting in loss-of-function of the gene. Authors performed in silico analysis on the 115 re-sequenced cucumber accessions and identified 31 accessions carrying the TE either homozygously or heterozygously. Of the 31 accessions, 29 are present in our CG population (Supplementary File S7), which showed a significant reduced DI compared to lines without the TE insertion (Figure 10b). The *CsaMLO8* gene (Chr5: 24,827,408…24,831,456 bp) is next to the QTL *pmG5.4* (Figure 4), indicating that the mutant *CsaMLO8* allele may explain the effect of this QTL, as it was the case for colocalized QTLs of this region in the study of He et al. [17] and Nie et al. [18,23]. Next to the *CsaMLO8* gene, the GATA transcription factor gene *CsaM622830* (Chr.5: 24735193…24738505) is also nearby *pmG5.4* (Figure 4). The *CsaM622830* gene is reported as the candidate gene for PM and DM resistance derived from *Cucumis hystrix* [25]. SNPs presented in *CsaM622830* were analyzed and two haplotypes with alternate SNPs showed significant DI difference only on PM (Supplementary File S7, Figure 10b). SNPs in both the *CsaMLO8* and *CsaM622830* gene showed no significant effect on the DIs of DM (Figure 10b).

Additionally, the *CsGy5G015660* gene on chromosome 5 (*Csa5G464830*, Chr5: 16,288,582…16,292,061 based on the Chinese Long genome v2) was reported as a candidate gene for PM resistance derived from a Korean cucumber inbred line [16]. Natural variation of *CsGy5G015660* alleles was observed using 115 core germplasm, which, however, does not lead to significant DI different between the CG lines carrying contrast haplotypes (Supplementary File S7, Figure 10b).

## 4. Discussion

The inheritance of PM resistance is complicated in cucumber. Few studies reported that PM resistance was controlled by a recessive gene [9,10]. Most studies showed that PM resistance was a quantitative trait that controlled by multiple genes and likely recessively inherited in cucumber [11,12,16,17,18,19,21,25,35]. In agreement with previous studies, the results showed that PM resistance of the 94 CG lines was controlled by 13 QTLs scattered over six of the seven cucumber chromosomes.

With the resequencing data of the CG lines, SNPs of candidate genes in the QTL intervals could be studied leading to identification of genes of which CG lines carrying alternate SNPs showed significant DI difference. In most case, the majority (if not all) of the 21 HS line shared one haplotype, while only 3 to 8 of the HR lines had the alternate haplotype (Figure 5, Figure 6, Figure 7, Figure 8 and Figure 9). The latter could indicate that the HR lines were not truly resistant. This may be explained by the fact that natural infection in the fields was applied in this study. With natural infection, the uniformity might not be guaranteed because of the influence of environmental conditions of different sites/years/seasons, pathogen species, and population in the field. Thus, resistance in some of the HR lines could be a false positive. Alternatively, it could be due to the possibility that PM races present in difference seasons were not the same. If the resistance conferred by individual QTLs was race-specific, some QTLs would be detected only in specific experiments. While, for the HS lines, it was more accurate since a line HS had a DI above 50 in at least two of the four experiments (Figure 1). To further confirm that resistance in the HR lines as well as CG lines with intermediate resistance, disease assays with an artificial PM infection should be applied to confirm the resistance in HR lines.

In the intervals of the mapped stable and novel QTLs, several candidate genes were predicated (Figure 5, Figure 6, Figure 7, Figure 8 and Figure 9). Of the eight candidate genes (Table 2), only one candidate gene for *pmG4.1* belongs to a typic resistance (R) gene encoding a receptor-like kinase (RLK), which is well-known to be involved in disease resistance to biotrophic pathogens [36]. The rest of the candidate genes have not been shown to have a direct link with disease resistance in plants. It is intriguing that none of canonical dominant R genes, encoding either a RLK or a nucleotide-binding site (NBS)-leucine-rich repeat (LRR) protein, have been described for PM resistance in cucumber. This may be related to the facts that (1) cucumber genome contains a small NBS-LRR gene family [37,38], and (2) the PM resistance in cucumber is mostly quantitatively or recessively inherited. 

Further, expected SNP haplotypes for the predicted candidate genes were more common in the HS lines than in the HR lines (Figure 5, Figure 6, Figure 7, Figure 8 and Figure 9). As discussed above, resistance in some HR lines might be a false positive or race-specific. Thus, it is essential to confirm the resistance in the HR lines by artificial infection with specific PM races. In addition, we only studied the SNPs in CDS regions. SNPs in promoter and intron regions should be further evaluated in order to verify the candidate genes for their effect on PM resistance in cucumber.

In cucumber, QTLs for PM resistance have previously been detected on chromosome 5 in an interval where the *CsaMLO8* gene is located [10,11,16,17,18,19,20,21,22,24,25]. In the CG population, the QTL *pmG5.4* is colocalized with *CsaMLO8*, and the CG lines carrying the mutant allele (with TE insertion) of *CsaMLO8* [28] showed a significant reduction of disease symptoms. Further, SNPs in the GATA transcription factor gene *Csa5M622830* [25] also contribute significantly to the DI difference between the two haplotypes. Since the *CsaMLO8* gene is closely located with *pmG5.4*, we argue that the mutant allele of *CsaMLO8* may be the major contributor to this QTL for the hypocotyl resistance to PM in cucumber. Hypocotyl resistance was shown to be needed for a ‘complete’ or ‘leaf resistance’ in cucumber [7]. The TE-insertion in the mutant allele of *CsaMLO8* was also found in an Indian accession PI 215,589 of *C. sativus* var. *hardwickii*, suggesting that PI 215,589 may be the donor of the hypocotyl PM resistance [28].

**Figure 11:**
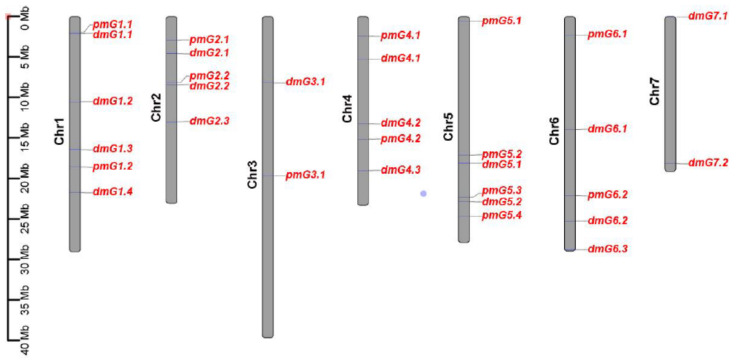
Locations of QTLs identified in the CG population for powdery mildew (PM, this study) and downy mildew (DM resistance (our previous study, [35]).

The *MLO* genes represent a well-studied example of plant S genes for susceptibility to different PM species across different plant species [1,27]. In addition to the *MLO* genes, *PMR* genes are also shown to be S genes for PM, including *PMR5* and *PMR6* [28]. The recessively inherited resistance to PM in cucumber indicates that the resistance is governed by impaired S genes, such as the *MLO* and *PMR* genes [24,26,28,29]. Analyzing SNPs in cucumber orthologues of the *PMR5* and *PMR6* genes, showed that CG lines carrying contrasting SNP haplotypes of the *CsPMR6-10* gene had a significant DI difference. Thus, in addition to the *Csa5G488800* gene, *CsPMR6-10* might also be the candidate gene for the QTL *pm5.2*. In a review, Schouten et al., (2014) mapped a set of cucumber orthologues of plant S-genes reported for PM and DM [27]. As we demonstrated for the *MLO* and *PMR* genes, allelic variants of these cucumber S genes could be identified and their effect on PM and DM resistance/susceptibility may be discovered with the disease phenotypes of the CG lines for PM (this study) and for DM [34]. Further, colocalization of these S genes with PM/DM QTLs mapped in other studies (Table 1) may indicate that mutations in the colocalized S-genes contribute to the QTL effect, as it is the case for the mutant allele of *CsaMLO8* in this study as well as other studies [23,27,28]. In cucumber, the resistance to DM and PM has been often found in one donor accession, such as in the PR37 line and the accession PI 197,088 (Table 1) [12,14,15,21,22]. In this CG population, a significant association between resistances to PM and DM was observed (Supplementary Figure S2). We detected 13 loci for PM resistance in this study and 18 loci for DM resistance in our previous study [34]. A number of loci are colocalized, including *pmG1.1* and *dmG1.1*, *pmG2.2* and *dmG2.2*, *pmG5.2*, and *dmG5.1*, as well as *pmG5.3* and *dmG5.2* (Figure 11). The significant association of DM and PM resistance in this CG population may be due to the linked genes located in the same chromosomal region or the pleotropic effect of the same gene. Our study on three cloned candidate genes *CsaMLO8*, *CsaM622830*, and *CsPMR6-10* showed that they contributed to only PM resistance in this CG population (Figure 10). Therefore, we speculate that the linked genes each with effect either on PM or DM might be the cause for the significant correlation between PM and DM resistance in this CG population, although the pleotropic effect of the same gene for resistance to both pathogens could not be ruled out.

## 5. Conclusions

In our cucumber CG germplasm, resistance to both PM (this study) and DM resistance [34] was identified, which are associated with many genetic loci scattered all over the seven cucumber chromosomes. Some loci are located in chromosomal regions where QTLs and plant S gene for PM and DM have been reported in previous studies. For PM resistance, we showed in this study that previously cloned candidate genes contributed to PM resistance in this CG population, including *CsaMLO8*, *CsaM622830*, and *CsPMR6-10*. While, the DM resistance could not be attributed to the previously cloned genes, including *Sgr*, *CsLRK10L2*, and *CsAAP2A* [34]. Some QTLs for DM and PM are clustered together in certain chromosomal regions, which supported the significant correlation between PM and DM resistance in this CG germplasm. Since resistance to both DM and PM was often described with a recessive inheritance, it is likely that the resistance may be conferred by loss of function in plant S genes, such as the *MLO* and *PMR* genes. With the availability of the sequenced genomes of these CG lines, allele mining could be performed for reported plant S genes [39]. Further, the association between allelic variants of these S genes with colocalized QTLs could be evaluated. Such studies will enable to explore the CG germplasm for using their natural mutant alleles of certain S genes in breeding cucumber with resistance to both PM and DM.

## Figures and Tables

**Table 2:** Overview of predicted cucumber genes in the mapped genetic loci for powdery mildew resistance in this study.

Genetic Locus	Gene Number	Predicted Protein
*pmG2.1*	*Csa2G030010-*	DNA polymerase
	*Csa2G030020*	a Lin-9-like protein
	*Csa2G030030*	an unknown protein
*pmG3.1*	*Csa3G414050*	Unknown protein
*pmG4.1*	homolog of *Csa4G022350*	Leucine-rich repeat protein kinase family protein
*pmG5.2*	*Csa5G488800*	RNA recognition motif
*pmG5.3*	*Csa5G603950*	Pyruvate dehydrogenase E1 component α subunit
	*Csa5G603960*	Paired amphipathic helix protein Sin3

## Data Availability

Not applicable.

## Supplementary Materials

The following are available online at https://www.mdpi.com/article/10.3390/genes12040584/s1, Figure S1: Frequency distribution and Spearman rank correlations of mean PM disease scores in CG population at three rating times with two criteria under three environments. *** indicates significance at *p* < 0.001. Figure S2: Frequency distribution and Spearman rank correlations of mean PM and DM disease scores in CG population at three rating times with two criteria under three environments. *, **, *** indicate significance at *p* < 0.05, *p* < 0.01, *p* < 0.001, respectively. Supplementary File S1: Accession numbers, origin, ecotypes, and disease index of the CG lines. Supplementary File S2: The disease index of different market types. Supplementary File S3: The selected highly resistant (HR), intermediate resistant (IR) and highly susceptible (HS) line. Supplementary File S4: Comparison of loci identified by GWAS with previously reported QTLs. Supplementary File S5: SNP haplotypes of the selected highly resistant and susceptible lines. Supplementary File S6: SNP haplotypes of cucumber orthologues of the *PMR5* and *PM6* genes and the disease index of PM infection in different experiments of this study. Supplementary File S7: SNP haplotypes of the *CsaMLO8*, *CsaM622830*, and *CsGy5G015660* genes and the disease index of PM infection in different experiments of this study.

## References

[1] Pérez-García A., Romero D., Fernández-Ortuño D., López-Ruiz F., De Vicente A., Tores J.A. (2009). The powdery mildew fungus *Podosphaera fusca* (synonym *Podosphaera xanthii*), a constant threat to cucurbits. Mol. Plant Pathol..

[2] Ishii H., Fraaije B.A., Sugiyama T., Noguchi K., Nishimura K., Takeda T., Amano T., Hollomon D.W. (2001). Occurrence and Molecular Characterization of Strobilurin Resistance in Cucumber Powdery Mildew and Downy Mildew. Phytopathology.

[3] Kooistra E. (1968). Powdery mildew resistance in cucumber. Euphytica.

[4] Panstruga R., Schulze-Lefert P. (2002). Live and let live: Insights into powdery mildew disease and resistance. Mol. Plant Pathol..

[5] Zijlstra S., Groot S.P. (1992). Search for novel genes for resistance to powdery mildew (*Sphaerotheca fuliginae*) in cucumber (*Cucumis sativus*). Euphytica.

[6] Block C.C., Reitsma K.R. (2005). Powdery Mildew Resistance in the U.S. National Plant Germplasm System Cucumber Collection. HortScience.

[7] Shanmugasundaram S., Williams P., Peterson C.J.P. (1971). Inheritance of resistance to powdery mildew in cucumber. Phytopathology.

[8] Clair D.A.S. (2010). Quantitative Disease Resistance and Quantitative Resistance Loci in Breeding. Annu. Rev. Phytopathol..

[9] Liu L., Yuan X., Cai R., Pan J., Zhu L. (2008). Quantitative Trait Loci for Resistance to Powdery Mildew in Cucumber under Seedling Spray Inoculation and Leaf Disc Infection. J. Phytopathol..

[10] Liu P., Miao H., Lu H., Cui J., Tian G., Wehner T., Gu X., Zhang S. (2017). Molecular mapping and candidate gene analysis for resistance to powdery mildew in *Cucumis sativus* stem. Genet. Mol. Res..

[11] Zhang S., Liu M., Miao H., Zhang S., Yang Y., Xie B., Gu X. (2011). QTL mapping of resistance genes to powdery mildew in cucumber (*Cucumis sativus* L.). Sci. Agric. Sin..

[12] Sakata Y., Kubo N., Morishita M., Kitadani E., Sugiyama M., Hirai M. (2006). QTL analysis of powdery mildew resistance in cucumber (*Cucumis sativus* L.). Theor. Appl. Genet..

[13] Wilson J., John C., Wohler H., Hoover M. (1956). Two foreign cucumbers resistant to bacterial wilt and powdery mildew. Plant Dis. Rep..

[14] Smith P. (1948). Powdery Mildew Resistance in Cucumber.

[15] Barnes W., Epps W. (1956). Powdery mildew resistance in South Carolina cucumbers. Plant Dis. Rep..

[16] Zhang C., Anarjan M.B., Win K.T., Begum S., Lee S. (2021). QTL-seq analysis of powdery mildew resistance in a Korean cucumber inbred line. Theor. Appl. Genet..

[17] He X., Li Y., Pandey S., Yandell B.S., Pathak M., Weng Y. (2013). QTL mapping of powdery mildew resistance in WI 2757 cucumber (*Cucumis sativus* L.). Theor. Appl. Genet..

[18] Nie J., He H., Peng J., Yang X., Bie B., Zhao J., Wang Y., Si L., Pan J.-S., Cai R. (2015). Identification and fine mapping of *pm5.1*: A recessive gene for powdery mildew resistance in cucumber (*Cucumis sativus* L.). Mol. Breed..

[19] De Ruiter W., Hofstede R., de Vries J., Van den Heuvel H., Pitrat M. (2008). Combining QTLs for resistance to CYSDV and powdery mildew in a single cucumber line. Cucurbitaceae.

[20] Zhang H., Wang Z., Mao A., Zhang F., Wang Y., Xu Y. (2008). SSR markers linked to the resistant gene of cucumber powdery mildew. Acta Agri Boreali Sin..

[21] Wang Y., Vandenlangenberg K., Wen C., Wehner T.C., Weng Y. (2018). QTL mapping of downy and powdery mildew resistances in PI 197088 cucumber with genotyping-by-sequencing in RIL population. Theor. Appl. Genet..

[22] Fukino N., Yoshioka Y., Sugiyama M., Sakata Y., Matsumoto S. (2013). Identification and validation of powdery mildew (*Podosphaera xanthii*)-resistant loci in recombinant inbred lines of cucumber (*Cucumis sativus* L.). Mol. Breed..

[23] Nie J., Wang Y., He H., Guo C., Zhu W., Pan J., Li D., Lian H., Pan J., Cai R. (2015). Loss-of-function mutations in *CsMLO1* confer durable powdery mildew resistance in cucumber (*Cucumis sativus* L.). Front. Plant Sci..

[24] Xu X., Yu T., Xu R., Shi Y., Lin X., Xu Q., Qi X., Weng Y., Chen X. (2016). Fine mapping of a dominantly inherited powdery mildew resistance major-effect QTL, *Pm1.1*, in cucumber identifies a 41.1 kb region containing two tandemly arrayed cysteine-rich receptor-like protein kinase genes. Theor. Appl. Genet..

[25] Zhang K., Wang X., Zhu W., Qin X., Xu J., Cheng C., Lou Q., Li J., Chen J. (2018). Complete resistance to powdery mildew and partial resistance to downy mildew in a *Cucumis hystrix* introgression line of cucumber were controlled by a co-localized locus. Theor. Appl. Genet..

[26] Pavan S., Jacobsen E., Visser R.G.F., Bai Y. (2009). Loss of susceptibility as a novel breeding strategy for durable and broad-spectrum resistance. Mol. Breed..

[27] Schouten H.J., Krauskopf J., Visser R.G.F., Bai Y. (2014). Identification of candidate genes required for susceptibility to powdery or downy mildew in cucumber. Euphytica.

[28] Berg J.A., Appiano M., Martínez M.S., Hermans F.W.K., Vriezen W.H., Visser R.G.F., Bai Y., Schouten H.J. (2015). A transposable element insertion in the susceptibility gene *CsaMLO8* results in hypocotyl resistance to powdery mildew in cucumber. BMC Plant Biol..

[29] Van Schie C.C., Takken F.L. (2014). Susceptibility genes 101: How to be a good host. Annu. Rev. Phytopathol..

[30] Qi J., Liu X., Shen D., Miao H., Xie B., Li X., Zeng P., Wang S., Shang Y., Gu X. (2013). A genomic variation map provides insights into the genetic basis of cucumber domestication and diversity. Nat. Genet..

[31] Zhang S.P., Liu M.M., Miao H., Yang Y.H., Xie B.Y., Wehner T.C., Gu X.F. (2013). Chromosomal Mapping and QTL Analysis of Resistance to Downy Mildew in *Cucumis sativus*. Plant Dis..

[32] SAS Institute (2012). SAS/OR 9.3 User’s Guide: Mathematical Programming Examples.

[33] Purcell S., Neale B., Todd-Brown K., Thomas L., Ferreira M.A., Bender D., Maller J., Sklar P., de Bakker P.I., Daly M.J. (2007). PLINK: A Tool Set for Whole-Genome Association and Population-Based Linkage Analyses. Am. J. Hum. Genet..

[34] Liu X., Lu H., Liu P., Miao H., Bai Y., Gu X., Zhang S. (2020). Identification of Novel Loci and Candidate Genes for Cucumber Downy Mildew Resistance Using GWAS. Plants.

[35] Wang Y., Bo K., Gu X., Pan J., Li Y., Chen J., Wen C., Ren Z., Ren H., Chen X. (2020). Molecularly tagged genes and quantitative trait loci in cucumber with recommendations for QTL nomenclature. Hortic. Res..

[36] Zhou Z., Pang Z., Zhao S., Zhang L., Lv Q., Yin D., Li D., Liu X., Zhao X., Li X. (2019). Importance of *OsRac1* and *RAI1* in signalling of nucleotide-binding site leucine-rich repeat protein-mediated resistance to rice blast disease. New Phytol..

[37] Wan H., Yuan W., Bo K., Shen J., Pang X., Chen J. (2013). Genome-wide analysis of NBS-encoding disease resistance genes in *Cucumis sativus* and phylogenetic study of NBS-encoding genes in Cucurbitaceae crops. BMC Genom..

[38] Huang S., Li R., Zhang Z., Li L., Gu X., Fan W., Lucas W.J., Wang X., Xie B., Ni P. (2009). The genome of the cucumber, *Cucumis sativus* L.. Nat. Genet..

[39] Berg J.A., Hermans F.W.K., Beenders F., Lou L., Vriezen W.H., Visser R.G.F., Bai Y., Schouten H.J. (2020). Analysis of QTL DM4.1 for Downy Mildew Resistance in Cucumber Reveals Multiple subQTL: A Novel *RLK* as Candidate Gene for the Most Important subQTL. Front. Plant Sci..

