# Supplementary material for: Identification of Novel Loci and Candidate Genes for Resistance to Powdery Mildew in a Resequenced Cucumber Germplasm
Source: Genes (Basel). 2021 Apr 16;12(4):584. doi: 10.3390/genes12040584 (PMC8072792; doi:10.3390/genes12040584)
Supplement: Supplementary file 1 [file genes-12-00584-s001.zip › genes-1137498-supplementary/Supplemenrary file4.4/2021-02-20 Supplementary file 4_SNPs and overlap with other study.docx]

**Supplementary file 4.** Comparison of loci identified by GWAS with previously reported QTLs.

| **Locus** | **SNP name** | **Chromosome** | **SNP Position**  **(Mb)** | **Flanking markers** | | **Physical location (Mb)** | **Reference** |
| --- | --- | --- | --- | --- | --- | --- | --- |
| *pmG-1.1* | SNP28206 | 1 | 1.95 | SSR23757 | | 1.92 | Zhang et al. 2013 |
|  |  |  |  | UW019729 | | 5.37 |  |
| *pmG-1.2* | SNP337008 | 1 | 18.61 | SSR04805 | | 17.06 | He et al. 2013 |
|  |  |  |  | SSR084288 | | 29.17 |  |
| *pmG-2.1* | SNP597707 | 2 | 2.91 | Novel | |  |  |
| *pmG-2.2* | SNP723128 | 2 | 8.39 | Marker2_8520308 | | 8.52 | Wang et al.2018 |
|  |  |  |  | Marker2_12912775 | | 12.91 |  |
| *pmG-3.1* | SNP1353661 | 3 | 19.68 | Novel | |  |  |
|  |  |  |  |  |  |  |  |
| *pmG-4.1* | SNP1730726 | 4 | 2.43 | Novel | |  |  |
| *pmG-4.2* | SNP1978726 | 4 | 15.16 | SSR06 | | 9.72 | He et al. 2013 |
|  |  |  |  | SSR16038 | | 20.40 |  |
| *pmG-5.1* | SNP2149033 | 5 | 0.64 | CSWTA04 | | 3.71 | Fukino et al. 2013 |
|  |  |  |  | CSJCT315 | | 7.02 |  |
| *pmG-5.2* | SNP2452755 | 5 | 17.11 | SSR15893 | | 15.27 | He et al. 2013 |
|  |  |  |  | UW013295 | | 21.33 |  |
| *pmG-5.3* | SNP2522699 | 5 | 22.31 | uw013295 | | 21.41 | He et al. 2013 |
|  |  |  |  | SSR13237 | | 25.17 |  |
| *pmG-5.4* | SNP2563088 | 5 | 24.67 | Marker5_24647715 | | 24.64 | Wang et al.2018 |
|  |  |  |  | Marker5_26224508 | | 26.22 |  |
| *pmG-6.1* | SNP2670013 | 6 | 3.12 | SSR7198 | | 5.26 | Fukino et al. 2013 |
|  |  |  |  | CS41 | | - |  |
| *pmG-6.2* | SNP3057280 | 6 | 22.11 | Marker6_23875073 | 23.87 | | Wang et al.2018 |
|  |  |  |  | Marker6_26499531 | 26.49 | |  |
