# Supplementary figures and images for: Identification of Novel Loci and Candidate Genes for Resistance to Powdery Mildew in a Resequenced Cucumber Germplasm
Source: Genes (Basel). 2021 Apr 16;12(4):584. doi: 10.3390/genes12040584 (PMC8072792; doi:10.3390/genes12040584)

## Slide 1
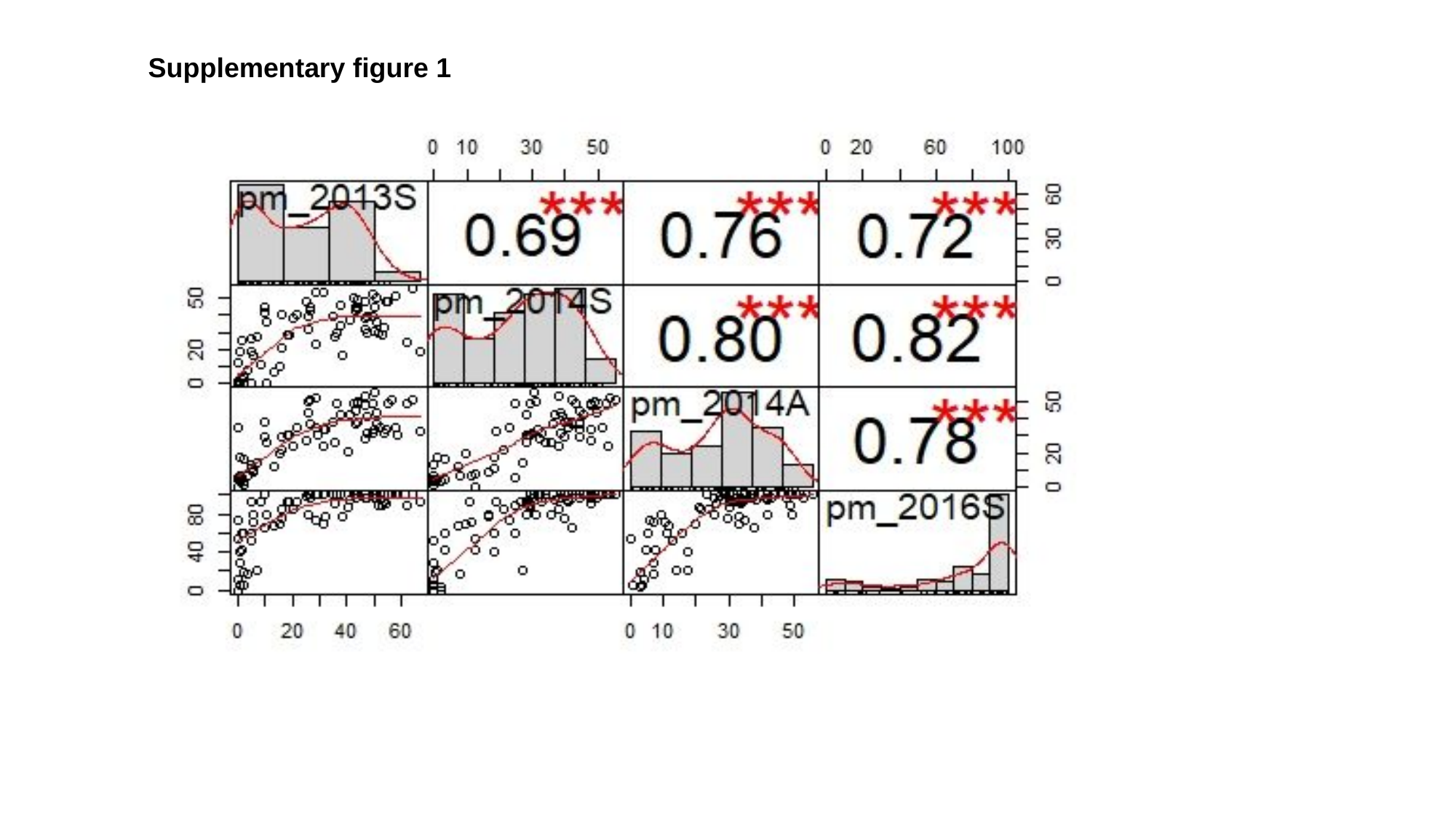

Supplementary figure 1

## Slide 2
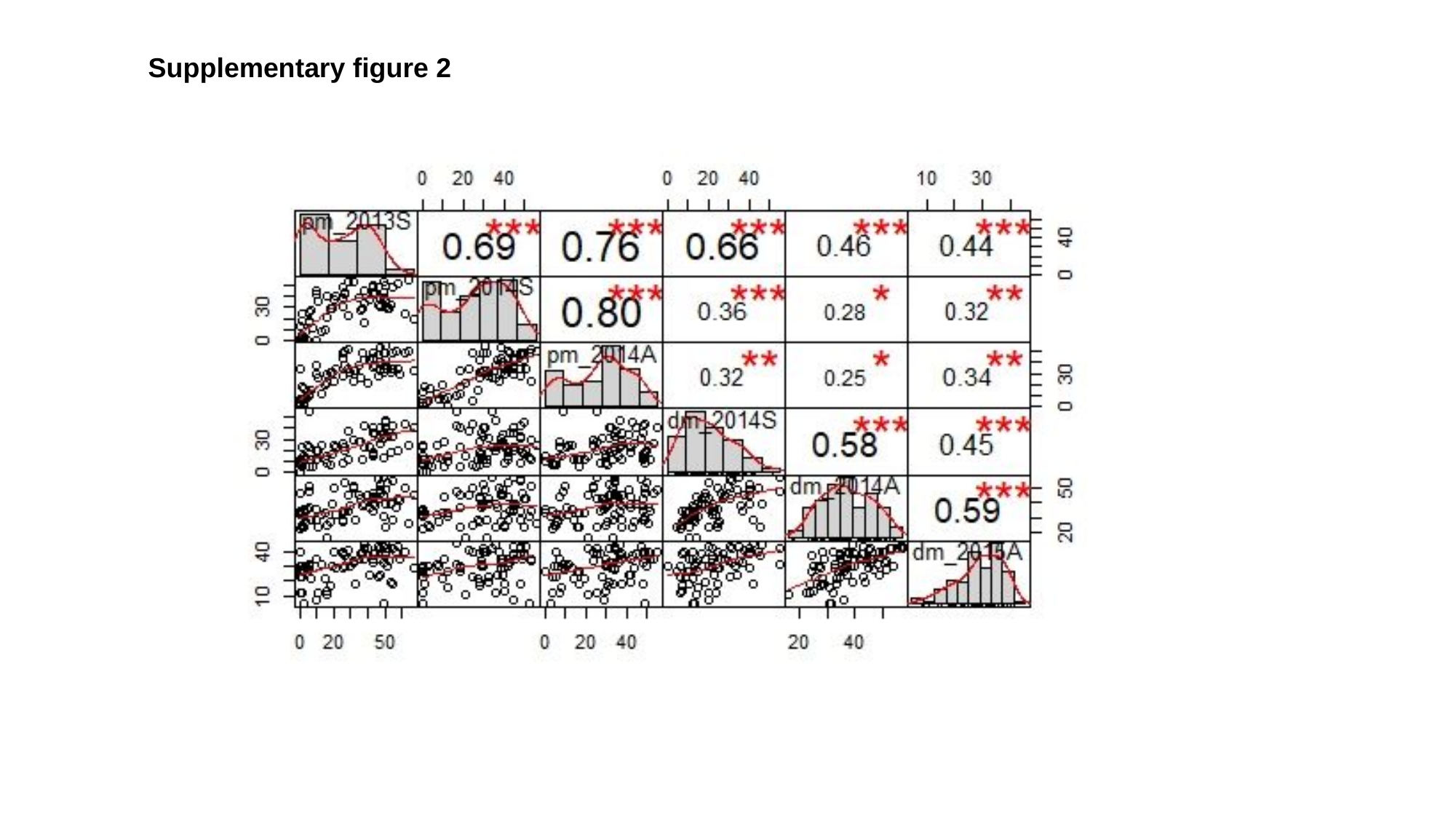

Supplementary figure 2

Supplement: Supplementary file 1 [file genes-12-00584-s001.zip › genes-1137498-supplementary/Supplemenrary file4.4/2021-02-20 Supplementary Figure.pptx]
